# The Role of Lung Microbiome in Fibrotic Interstitial Lung Disease—A Systematic Review

**DOI:** 10.3390/biom14030247

**Published:** 2024-02-20

**Authors:** Ruxandra Puiu, Nicoleta Stefania Motoc, Sergiu Lucaciu, Maria Victoria Ruta, Ruxandra-Mioara Rajnoveanu, Doina Adina Todea, Milena Adina Man

**Affiliations:** 1 Department of Medical Sciences, Pulmonology, Faculty of Medicine, Iuliu Hatieganu University of Medicine and Pharmacy, 400012 Cluj-Napoca, Romania or puiu.ruxandra@elearn.umfcluj.ro (R.P.); lucaciuserju@gmail.com (S.L.); dtodea@umfcluj.ro (D.A.T.); manmilena50@yahoo.com (M.A.M.); 2 I Department of Pulmonology, “Leon Daniello” Clinical Hospital of Pulmonology, 400371 Cluj-Napoca, Romania; victoria.sute@yahoo.com; 3 Department of Palliative Medicine, Faculty of Medicine, Iuliu Hatieganu University of Medicine and Pharmacy, 400012 Cluj-Napoca, Romania; ruxandra.rajnoveanu@umfcluj.ro

**Keywords:** microbiome, interstitial lung disease, fibrosis, lung, progressive

## Abstract

Interstitial Lung Disease (ILD) involves lung disorders marked by chronic inflammation and fibrosis. ILDs include pathologies like idiopathic pulmonary fibrosis (IPF), connective tissue disease-associated ILD (CTD-ILD), hypersensitivity pneumonitis (HP) or sarcoidosis. Existing data covers pathogenesis, diagnosis (especially using high-resolution computed tomography), and treatments like antifibrotic agents. Despite progress, ILD diagnosis and management remains challenging with significant morbidity and mortality. Recent focus is on Progressive Fibrosing ILD (PF-ILD), characterized by worsening symptoms and fibrosis on HRCT. Prevalence is around 30%, excluding IPF, with a poor prognosis. Early diagnosis is crucial for optimizing outcomes in PF-ILD individuals. The lung microbiome comprises all the microorganisms that are in the respiratory tract. Relatively recent research try to evaluate its role in respiratory disease. Healthy lungs have a diverse microbial community. An imbalance in bacterial composition, changes in bacterial metabolic activities, or changes in bacterial distribution within the lung termed dysbiosis is linked to conditions like COPD, asthma and ILDs. We conducted a systematic review of three important scientific data base using a focused search strategy to see how the lung microbiome is involved in the progression of ILDs. Results showed that some differences in the composition and quality of the lung microbiome exist in ILDs that show progressive fibrosing phenotype. The results seem to suggest that the lung microbiota could be involved in ILD progression, but more studies showing its exact pathophysiological mechanisms are needed.

## 1. Introduction

Interstitial lung disease (ILD) refers to a group of heterogeneous lung disorders characterized by chronic inflammation and fibrosis of the lung tissue [1]. Extensive scholarly literature on ILD has explored its various subtypes, including idiopathic pulmonary fibrosis (IPF), connective tissue disease-associated ILD (CTD-ILD), and hypersensitivity pneumonitis (HP), among others [2,3]. Research has focused on understanding the pathogenesis [4], clinical presentation, diagnostic approaches, and treatment options [5] for ILD. The pathogenesis of IPF has been extensively researched. Studies have identified several pathophysiological mechanisms that are believed to contribute to the development and progression of IPF. These mechanisms include epithelial injury, fibroblast activation, inflammatory mediators, genetic predisposition, oxidative stress, and microenvironmental changes [6]. High-resolution computed tomography (HRCT) plays a crucial role in the diagnosis and classification of ILD, enabling the identification of specific radiological patterns [1]. Biomarkers [7,8] and genetic factors [9] have been investigated to improve diagnostic accuracy, predict disease progression, and guide treatment decisions. Therapeutic options for ILD include pharmacological interventions, such as antifibrotic agents for IPF, immunosuppressive therapies for CTD-ILD, and avoidance of offending antigens in HP, as well as supportive measures such as oxygen supplementation and pulmonary rehabilitation [10,11]. Despite advances, ILD remains a challenging condition with significant morbidity and mortality, necessitating ongoing research to enhance our understanding and improve patient outcomes.

A new emerging concept has taken the attention of researchers in the last few years. Progressive fibrosing interstitial lung disease (PF-ILD) is defined as worsening respiratory symptoms, lung function, and extent of fibrosis on high-resolution computed tomography [12]. The prevalence of this form of ILD in approximately 30% for all types of ILD other than IPF [13,14]. The most concerning aspect of PF-ILD presents with poor prognosis similar to IPF [15]. ILDs that can progress to becoming PF-ILD are non-autoimmune-related (50.0%), autoimmune-related (40.0%) and sarcoidosis (10.0%) [16]. Several risk factors have been studied for developing PF-ILD [17]. Management of progressive fibrosing ILD typically involves a multidisciplinary approach, including pharmacological interventions such as antifibrotic medications, supportive care, and in some cases, lung transplantation. Early diagnosis and appropriate management are crucial for optimizing outcomes in individuals with progressive fibrosing ILD [18].

The lung microbiome refers to the collection of microorganisms residing in the respiratory tract, challenging the long-held belief that the lungs are sterile. Emerging research has focused on characterizing the lung microbiome and understanding its role in respiratory health and disease. The terms used surrounding the microbiome and relevant for our study are described in Table 1 as described from other sources [19,20].

The analysis of the lung microbiome involves the application of diverse methodologies to elucidate the composition and functional attributes of microbial communities within the respiratory tract. Key methodologies include Next-Generation Sequencing (NGS), which encompasses 16S rRNA Gene Sequencing for targeted sequencing of the 16S ribosomal RNA gene, enabling taxonomic identification and classification of bacterial constituents. Metagenomic Sequencing, a comprehensive approach, entails sequencing the entire genomic content of a sample, facilitating the identification of bacterial, viral, and fungal entities.

Quantitative Polymerase Chain Reaction (qPCR), with a focus on the 16S rRNA gene, enables the quantification of specific bacterial taxa, offering insights into the relative abundance of select microbial populations. Other testing techniques for lung microbiome include microbiome microarrays, Fluorescence In Situ Hybridization (FISH), shotgun metagenomics, functional metagenomics, multi-omics approaches and bioinformatics analysis [21,22].

Sampling methodologies employed for culture-independent analysis of the microbiota in the lower respiratory tract in both human and animal studies encompass various techniques. These approaches encompass specimens obtained through bronchoscopy, including bronchoalveolar lavage fluid (BALF) and protected specimen brushings, as well as samples derived from sputum, breath condensate, and surgically excised lung tissue. While bronchoscopy-acquired BALF stands out as the most prevalent method, it is noteworthy that there is no universally accepted gold standard in the field. Additionally, existing guidelines do not currently provide recommendations for microbiome sampling practices [23].

Studies have revealed a diverse microbial community in healthy lungs, with the predominance of bacteria such as *Streptococcus*, *Prevotella*, and *Veillonella* [24,25,26]. Imbalances in the lung microbiome, termed dysbiosis, have been associated with various lung conditions, including chronic obstructive pulmonary disease (COPD), asthma, cystic fibrosis (CF), and pneumonia [27,28,29]. Dysbiosis may involve shifts in microbial composition, decreased diversity, or overgrowth of potentially harmful bacteria. These microbial imbalances can influence local immune responses, airway inflammation, mucus production, and the development of respiratory symptoms [30,31]. Furthermore, interactions between the lung microbiome and the host immune system are thought to play a role in shaping lung health and disease progression [27,32]. However, the exact mechanisms by which the lung microbiome impacts respiratory health and disease remain a topic of ongoing investigation.

In this paper, we conducted a systematic review of available scientific research to investigate the role of the lung microbiome in interstitial lung disease progression, specifically in the development of PF-ILD. Previously, no research had demonstrated the microbiome as a potential factor in developing PF-ILD. Given the recent research regarding the lung microbiome’s implications in pulmonary pathology, we aimed to explore the possibility of the lung microbiome being a factor in the progression of fibrosis in ILD and the occurrence of PF-ILD.

## 2. Materials and Methods

**Figure 1:**
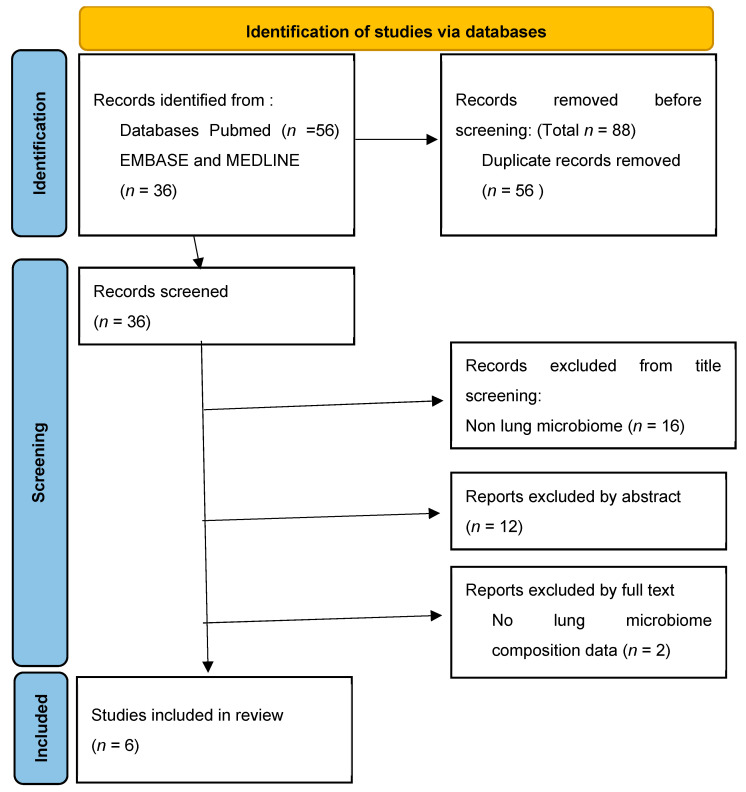
Identification of study selection via databases.

The question we wanted to answer in the writing of this systematic review was whether the lung microbiome is involved in the development and progression of interstitial lung disease. The study was planned, conducted and reported by the Preferred Reporting Items for Systematic Reviews and Meta-Analyses (PRISMA) (Figure 1) [33].

### 2.1. Search Strategy

Our search strategy was in a systematic matter using the scholarly literature from Embase, MEDLINE, and Pubmed. We searched for studies published from the 1 January 2000 to the 31 March 2023. The keywords we used were airway [all fields] OR lung [all fields] OR pulmonary [all fields] OR respiratory [all fields] AND microbiota [all fields] OR microbiome [all fields] AND interstitial lung disease [all fields] AND progression [all fields].

### 2.2. Study Selection

We included observational studies either prospective or retrospective, clinical trials and case reports which presented the lung microbiome from a quantitative and qualitative point of view from healthy patients and patients with any form of interstitial lung disease. From the interstitial lung disease family, we included research on idiopathic pulmonary fibrosis, hypersensitivity pneumonitis and sarcoidosis. We included studies that used either method of evaluating the bacterial load from culture based methods to new generational sequencing. The tissue of the sample did not make an exclusion criteria as long as it involved the lung. We then screened the available research for the ones that included development and progression biomarkers to correlate with the microbiome. We also considered the exacerbation of IPF as a form of progression of disease as its consequences have already been demonstrated. An additional search was carried out through the references of the included studies.

## 3. Results

Using the search strategy described earlier, we identified a total of 92 studies. After eliminating 56 studies due to duplication, we continued screening the research. We excluded 12 studies based on their title which did not match with the lung microbiome nor interstitial lung diseases. We then proceeded to analyze their text beginning with their abstract and we excluded 12 more studies. After reading the full text of the remaining 12 studies we eliminated 6 more. The reasons for eliminating the studies from the abstract and full text were they did not match the trial criteria: low enrolled participants, no details regarding the quantity or quality of lung bacteria, no correlations with biomarkers for the development or progression of ILD, ILD disease not regarding idiopathic pulmonary fibrosis, hypersensitivity pneumonitis and sarcoidosis.

### 3.1. Lung Microbiome in Idiopathic Pulmonary Fibrosis

#### 3.1.1. Lung Microbiome in Stable IPF

IPF is a chronic and progressive lung disease characterized by inflammation and ultimately the formation of fibrosis in the lungs. The exact cause of IPF is unknown, hence the term “idiopathic”, but it is believed to involve complex interactions between genetic predisposition, environmental factors, and aberrant wound healing responses in the lungs.

Clinical manifestations include progressive dyspnea, a persistent dry cough, and exercise intolerance. HRCT scans often reveal characteristic lung fibrosis patterns. A definitive diagnosis requires excluding other causes through a comprehensive evaluation, including clinical history, radiological findings, and, if needed, histopathological examination [34].

IPF has a poor prognosis, with a median survival of 3–5 years. Disease progression varies, and acute exacerbations can accelerate deterioration. Research has identified several key pathogenic mechanisms in IPF. Dysregulated wound healing, fibroblast activation, inflammation, oxidative stress, and impaired tissue repair contribute to fibrosis. Several mutations in specific genes have been associated with an increased risk of IPF [4,9].

While various treatments aim to slow progression and alleviate symptoms, no cure exists. Antifibrotic drugs like pirfenidone and nintedanib show efficacy. Lung transplantation may be considered for advanced cases [35].

IPF is an active area of research, and ongoing studies aim to better understand its underlying mechanisms, refine diagnostic approaches, and develop novel therapeutic strategies. The identification of potential biomarkers, including molecular and genetic markers, may facilitate early diagnosis, prognostication, and personalized treatment approaches [36].

In recent years, researchers have become increasingly interested in the role of the lung microbiome in IPF. Emerging evidence suggests that alterations in the lung microbiome may contribute to the pathogenesis of IPF. Even more so, the lung microbiome can be related to the progression of the disease and may also provide a potential therapeutic target. Dysbiosis in the lung microbiome has been associated with increased inflammation and activation of fibrotic pathways in animal models of pulmonary fibrosis. Certain microbial components or metabolites may also directly stimulate fibroblast activation and collagen production, contributing to tissue remodeling and fibrosis. However, the precise mechanisms by which the lung microbiome contributes to IPF pathogenesis are still being investigated, and further research is needed to establish causality and understand the underlying mechanisms.

Several studies have shown that IPF patients have reduced microbial diversity and altered microbial composition in the lung, compared to healthy individuals or other lung diseases [37]. A study by Molyneaux et al. (2014) [29] found that IPF patients carried twice the bacterial burden compared with healthy controls and COPD patients. This was a prospective, monocenter, observational study conducted on 65 IPF patients, 17 COPD patients, 27 healthy controls. Results showed that IPF patients had decreased microbial diversity in the lung, with a significant reduction in the abundance of certain bacterial taxa and increase in bacteria such as *Veillonella*, Neisseria, *Streptococcus* and *Haemophillus* spp.

Molyneaux et al. used PCR amplification of the 16S rRNA genes to detect bacterial species in bronchoalveolar lavage (BAL) samples and lung tissues of IPF patients. The study also aimed to demonstrate the link between lung microbiota and progression of IPF. The authors found that an increased bacterial load was correlated with a more rapid progression and a higher mortality rate of IPF.

Molyneaux et al. (2014) proposed several mechanisms through which bacteria may contribute to the development and progression of IPF. They suggest that chronic bacterial infection or colonization in the lungs could lead to sustained inflammation and activation of fibrotic processes. Bacterial products, such as endotoxins or other microbial components, may directly stimulate lung fibrosis by triggering an aberrant immune response and promoting tissue remodeling.

The article highlights the potential interplay between bacteria and host genetics in IPF. Molyneaux et al. (2014) discuss genetic variations associated with susceptibility to IPF and how these genetic factors may influence the lung microbiome and the host response to bacterial pathogens. The atuhors found that a greater bacterial load was associated with the presence of the MUC5B s3570590 T allele.

The relationship between the lung microbiome and disease progression in IPF is an active area of research. Han et al. (2017) [36] aimed to investigate the role of the lung microbiome in the disease progression of idiopathic pulmonary fibrosis (IPF). The researchers conducted their analysis using data from the COMET study, which was a multicenter, longitudinal observational study of 55 patients with IPF.

The researchers collected bronchoalveolar lavage (BAL) samples from a cohort of 55 IPF patients and performed DNA sequencing to characterize the microbial composition in the lung.

The findings of the study revealed IPF patients had reduced microbial diversity, indicating a less complex and imbalanced microbial community in the lung. Moreover, specific bacterial taxa such as *Prevotella*, *Veillonella* and *Escherichia* spp. were found to be the most common in IPF patients. A major limitation of this study is the absence of a control group.

Furthermore, the study also examined the association between the lung microbiome and disease progression in IPF. They correlated the microbial profiles with clinical markers of disease progression defined by various outcomes such as death, lung transplantation need, occurence of an exarcerbation, relative declince in FVC (forced vital capacity) > 10% or reduction with >15% of diffusing capacity of the lung for carbon monoxide (DLCO). The researchers found that the presence of *Streptococcus* spp. Or *Staphylococcus* spp. were associated with a higher risk of disease progression and poorer outcomes in IPF patients. These associations suggested that the lung microbiome could potentially serve as a prognostic indicator for IPF.

The study provided valuable insights into the potential role of the lung microbiome in the pathogenesis and progression of IPF. The dysbiosis observed in the lung microbiome of IPF patients suggests that alterations in microbial communities could contribute to the development and worsening of lung fibrosis. However, further research is needed to determine the underlying mechanisms and causal relationships between the lung microbiome and IPF.

The study by Huang et al. (2017) [38] titled “Microbes Are Associated with Host Innate Immune Response in Idiopathic Pulmonary Fibrosis” aimed to investigate the relationship between microbial communities and the host innate immune response in IPF. They analyzed the microbiome from bronchoalveolar lavage samples from IPF patients using 16S rRNA sequencing technique correlated it host immune-response-related signaling pathways. The inflammatory response was assesed by analyzing in vitro fibroblast function and leukocytes phenotypes. IPF patients showed alterations in microbial diversity and composition, with certain bacterial taxa such as *Prevotella* and *Staphylococcus* being negatively correlated with inflamatoy responses. The study also revealed an association between the lung microbiome and the expression of genes involved in the host immune response, particularly those related to inflammation and tissue remodeling. These findings suggest that microbial communities may influence the activation of the innate immune system and subsequent fibrotic processes in IPF. However, the study’s observational nature means that causality cannot be established. Nevertheless, the study contributes to our understanding of the complex interplay between microbes and the host immune system in the context of IPF.

A more recent study conducted by O’Dwyer et al. [39] Used a more advanced DNA sequecing method for characterising the microbiome from BAL of patients included in COMET original study. Its main objective was to describe how the microbiome influences alveolar inflammation and fibrosis of the lung. They correlated the microbiome composition with cytokine measurements of human BALF in vivo. The results reconfirmed previous studies meaning a greater bacterial burden is associated with progression in IPF and lung dysbiosis was correlated with increased inflammation, pro-fibrotic signaling and faulty repair. O’Dwyer et al. also used in their study a murine model with bleomicyine-induced fibrosis without lung microbiota to analyze how the process of inflammation can modulate lung microbiota and vice versa. Results showed that lung bacteria participate in pathogenesis in animal models of pulmonary fibrosis providing the first causal evidence that the microbiome participates in the pathogenesis and mortality of fibrotic lung disease.

All the studies described before have used BAL specimens to characterize the lung microbiome. Kitsios et al. [40] conducted a study using lung tissue specimens from lower lobe subpleural spaces, where fibrosis was most present. They analyzed lung samples from 40 IPF patients with end stage IPF with 16S rARN technique and samples from 37 controls in a case-control study. The results showed, in contrast with the previous findings, a low bacterial signal in the lung tissues collected from end stage IPF patients, similar to negative controls. It is important to note that the study has certain limitations. Specifically, it is believed that subpleural lung regions that exhibit advanced honeycombing are not suitable for bacterial growth. Additionally, end-stage disease may not accurately reflect the underlying pathophysiological process. Finally, it is possible that the microbiome affects the airways in IPF not only within the alveoli.

#### 3.1.2. Lung Microbiome in Acute Exacerbation of IPF (AE-IPF)

Acute exacerbations of IPF are defined as acute respiratory worsening with no identifiable cause. They are characterized by rapid-onset dyspnea, decline in lung function, and new infiltrates on imaging. Several factors have been identified as potential risk factors for acute exacerbations, including older age, male gender, low forced vital capacity (FVC), radiographic honeycombing, and a history of gastroesophageal reflux disease. Respiratory infections and aspiration events have also been implicated as triggers for exacerbations. Acute exacerbations of IPF are associated with a high mortality rate, with survival rates ranging from 30% to 50% at one year [41].

Acute exacerbations of IPF have a poor prognosis, and identifying predictors of exacerbations and effective therapeutic strategies remain important areas of research [42]. Biomarkers, such as circulating proteins and genetic variants, are being investigated to improve risk stratification and guide treatment decisions [43]. For this reason, the study of the microbiome of patients experiencing AE-IPF is essential as the microbiome could turn out to be a trigger and/or therapeutic target.

Having this in mind, Molyneaux et al. compared lung microbiota from BAL of 15 patients with stable IPF with the microbiota of 20 patients experiencing AE-IPF. The results showed that patients with AE-IPF carried a higher load of bacteria (up to four times) than their stable IPF counterparts. It was also observed a much higher value for Proteobacteria such as *Campylobacter* spp. and *Stenotrophomonas* spp. and a decrease for *Veillonella* spp. in the group of patients with AE-IPF. Given the fact that Proteobacteria are mostly found in the digestive tract, the authors hypothesized aspiration as a cause for AE-IPF occurrences [44].

Table 2 provides a summary of the main results that were presented earlier.

### 3.2. Lung Microbiome in Other Interstitial Lung Disease

#### 3.2.1. Lung Microbiome in Hypersensitivity Pneumonitis

Hypersensitivity pneumonitis (HP) is a common form of ILD developed by a predisposed genetic patient in contact with a specific inhaled antigen [45]. Prolonged exposure to the antigen may result in a fibrotic remodeling of the lung parenchyma which may be indistinguishable radiographic and/or histological from IPF [46]. This shows that the mechanisms involved in fibrotic development are the same throughout different fibrotic ILD. However, HP and IPF share different prognosis and courses of treatment [47].

Invernizzi et al. conducted a study to characterize the microorganisms from the lower airways of patients with Chronic hypersensitivity pneumonitis (CHP) [48]. The prospective study enrolled patients with a diagnosis of CHP, IPF, and a group of controls. The microbiome was studied from BAL of patients using PCR sequencing techniques and 16s RNA method. Invernizii et al. discovered differences in microbial profiles in lower airways of CHP and IPF patients. The differences were at the phylum level, with Firmicutes being most prevalent in IPF whereas Proteobacteria percentage was higher in patients with CHP. The genus of bacteria was also distinct between the two categories with *Staphylococcus* burden being increased in CHP and Actinomyces and *Veillonella* increased in IPF. Higher bacterial load was found in the lower airways of patients with CHP compared to the controls but lower compared to the IPF patients. The study did not find any association between the bacterial load or the bacterial profile and survival in CHP patients.

#### 3.2.2. Lung Microbiome in Sarcoidosis

Sarcoidosis is a chronic inflammatory disorder that appears as a result of exposure to an unknown antigen in a genetically predisposed patient. Sarcoidosis’ histological hallmark is the noncaseating granuloma which most likely appears in the lung and mediastinal lymph nodes [49]. The pathogenesis of sarcoidosis is still poorly understood but recent studies suggest that an aberrant immune response could be involved [50]. Epidemiological and microbiological studies suggest that at least in a fraction of patients, microbes or their products may trigger the immune response leading to sarcoid granuloma formation [51]. In light of this information, it could be easily hypothesized that lung microbiome could be involved in the pathogenesis and progression of sarcoidosis.

Zimmermann et al. conducted the first study analyzing the bacterial burden of the lower airways of patients with sarcoidosis. The study was cross-sectional and enrolled 71 patients with sarcoidosis, 11 with IPF and 10 healthy controls. The microbiome was characterized from BAL using 16s RNA sequencing techniques. Results showed no α diversity differences between the groups but found that *Atopobium* spp. was the most predominant species in sarcoidosis group while *Fusobacterium* spp. was significantly more abundant than in the healthy control group [52]. Following this research, Clarke et al. conducted a study to characterize the bacterial, fungal and viral respiratory burden of patients with sarcoidosis in comparison with healthy individuals. In doing so, they analyzed samples of tissue biopsied from lymph nodes, spleen, Kveim reagent, and BAL and performed bacterial 16S and fungal internal transcribed spacer ribosomal RNA gene sequencing. It was the first study using a metagenomic approach combining bacterial and fungal sequence tag analysis, virome shotgun sequencing, and WGS. The study did not identify a single causative agent but identified several candidate agents as sarcoidosis enriched. These include the Cladosporiaceae fungal family and Corynebacterium bacterial taxa. The study presented limited concordance between tissue samples [53].

Recently, for a more focused approach, Kristel at al. conducted a study to describe the microbiota of patients with sarcoidosis in comparison to healthy controls. They also looked at how antimicrobial peptides (AMP) vary depending on the microbiome composition in sarcoidosis patients. They enrolled 35 healthy patients and 35 sarcoidosis patients and used oral wash and protected bronchiololavage (PBAL) as specimens. The microbiome was characterized using 16s RNA sequencing techniques and antimicrobial peptides were analyzed from PBAL by enzyme-linked immunosorbent assay (ELISA). Results showed a different diversity of bacteria between the two groups and AMP was significantly lower in sarcoidosis compared to controls [54]. These suggest that dysbiosis could be involved in sarcoidosis’ pathogenesis and progression but its exact correlation has still to be determined.

## 4. Discussion

The lung microbiome is a promising area of research. The traditional belief of the respiratory tract being sterile has been challenged. Advancements in technology, particularly high-throughput DNA sequencing, have allowed researchers to identify and study microbial communities in various body sites, including the lower respiratory tract. This has brought in new ways of looking at respiratory disease from a microbiome perspective.

ILD is an umbrella term for many respiratory pathologies. For most ILD diseases, their cause, triggers for progression, and efficient therapy methods have not yet been demonstrated [55]. These limitations surrounding these diseases, the low quality of life for patients suffering from ILDs, and their poor prognosis make ILD a crucial subject for research [56]. Additionally, the newly developed term ILD with the progressive fibrosing phenotype, which shows a very poor prognosis comparable with IPF, has raised the attention of research communities. Studies have focused on finding accurate and early diagnosis methods, prognostic factors and efficient management [57,58].

In this paper, we wanted to see if the progression of ILD could be influenced by the lung’s microbiome. There is growing evidence of the potential role of the lung microbiome in PF-ILD. As reviewed above, studies have shown that there are alterations in the composition and diversity of the lung microbiome in individuals with ILD, including those with a progressive fibrosing phenotype. Changes in the abundance of specific microbial taxa, termed dysbiosis, have been demonstrated. Dysbiosis may contribute to an aberrant immune response in the lungs, potentially contributing to inflammation and fibrosis seen in PF-ILD. The significant differences in the composition and abundance of the bacterial communities in the lungs of ILD patients that progress in fibrosing phenotype could provide insights into the pathophysiological mechanisms of the disease.

In the meantime, a new area of research is looking at the microbiome as a potential therapeutic target. This includes interventions such as antibiotics, probiotics, or other strategies aimed at restoring a more balanced microbial community in the lungs. Retrospective investigations have demonstrated favorable effects on clinical outcomes among individuals with IPF who received prophylactic azithromycin or doxycycline [59,60]. Furthermore, findings from a preliminary study indicated that a three-month course of co-trimoxazole (trimethoprim–sulfamethoxazole) treatment yielded enhancements in quality of life and mitigated lung function decline in patients with fibrotic ILDs compared to a placebo [61].

Worth mentioning to the subject of this paper is the gut-lung axis. This concept refers to a significant interplay between the microbiota of the gastrointestinal tract and the respiratory system. Studies have shown that dysbiosis in the gastrointestinal tract can influence the lung microbiome through chemical messengers [62]. These imbalances in gut microbiota have been correlated with lung dysbiosis and changes in immune responses in chronic respiratory diseases such and ILD [63,64].

The concept that the lung microbiome might be responsible for the development of PF-ILD is relatively new. It’s worth noting that although there is growing evidence suggesting a link between the lung microbiome and PF-ILD, the exact mechanisms and causal relationships are not yet fully understood. We believe that this research could lead to more in vivo studies on this subject, opening up a new direction for tackling this disease. The lung microbiome could also serve as a therapeutic target with significant clinical implications. The lung microbiome is highly dynamic, and its role in health and disease is complex.

## Figures and Tables

**Table 1:** Microbiome terminology and definitions.

Term	Definition
Microbiome	community of microorganisms, including commensal, symbiotic, and pathogenic ones, found in a particular environment or body space
Microbiota	collection of living microorganisms present in a certain environment
Metagenome	genetic information of the microbiota obtained through genetic sequencing, which is then analyzed, organized, and identified through computational tools with the help of previously known sequence databases’
16S rRNA gene	component of the 30S small subunit of prokaryotic ribosomes, used in molecular studies due to its extremely slow rate of evolution and the presence of both variable and constant regions;
Operational taxonomic unit (OTU)	clusters of similar 16S rRNA gene sequences, with each OTU representing a taxonomic unit of a bacteria family or genus, depending on the sequence similarity threshold;
Dysbiosis	an imbalance in the composition of the microbiota of a given niche, related to changes in local conditions;
Abundance	total number of bacteria individuals in a specific sample;
Richness	is the number of different species/OTUs in a specific sample;
α-diversity	a measure of diversity within a sample and is based on the relative abundance of taxa;
β-diversity	a measure of the differences between samples from different groups;

**Table 2:** Synthesis of main results.

Author and Year	Design of the Study	Sample Size	Microbiome Assessment	Sample	Main Findings
Molyneaux et. al. 2014 [29]	Monocenter, observational, longitudinal, prospective	IPF patients:65 COPD patients: 17 Healthy controls: 27	PCR amplification of the 16S rRNA gene	BAL from middle lobe or lingular segment	Increased bacterial load was correlated with a more rapid progression and mortality rate in IPF. A greater bacterial load was associated with the presence of the MUC5B s3570590 T allele. *Streptococcus* spp. Or *Staphylococcus* spp. In the composition of lung microbiome was strongly associated with disease progression.
Han et. al. 2017 [36]	Multicenter, Observational, Longitudinal, prospective	IPF patients: 55	DNA sequencing	BAL from middle lobe, Lung tissues from explants;	*Streptococcus* and *Staphylococcus* OTUs appear to be associated with higher risk of disease progression;
Huang et. al. 2017 [38]	Multicenter, Observational prospective	IPF patients: 68	PCR amplification of the 16S rRNA gene	BAL from middle lobe	*Prevotella* and *Staphylococcus* are negatively correlated with inflammatory responses;
O’Dwyer et al. 2019 [39]	Multicenter, Observational, prospective	IPF patients: 68	ddPCR (droplet digital PCR) for 16S rRNA gene	BAL from middle lobe	Greater bacterial burden is associated with progression in IPF and lung dysbiosis was correlated with increased inflammation, pro-fibrotic signaling and faulty repair;
Kitosis et al. 2018 [40]	Case-control	IPF patients end stage: 40 Controls: 37	PCR amplification of the 16S rRNA gene	Subpleural lung tissue samples with advanced honey combing	Low bacterial signal in the end stage lung similar to controls;
Molyneaux et al. 2017 [44]	Monocenter, Observational, prospective	AE-IPF patients: 20 Stable IPD pateints: 15	PCR amplification of the 16S rRNA gene	BAL from middle lobe	A higher load of bacteria (up to four times) carried AE-IPF patients than the stable IPF counterparts;
